# Experimental study on shear behavior of Polyurethane/water glass-filled planar rock joints

**DOI:** 10.1371/journal.pone.0326262

**Published:** 2025-07-08

**Authors:** Hao Yu, Richeng Liu, Dedong Xiao, Shuchen Li, Xiaochuan Han, Minghui Hu, Yiyang Wang

**Affiliations:** 1 State Key Laboratory of Intelligent Construction and Healthy Operation and Maintenance of Deep Underground Engineering, China University of Mining and Technology, Xuzhou, China; 2 Shandong Hi-Speed Infrastructure Construction Co., Ltd., Jinan, China; University of Vigo, SPAIN

## Abstract

Rock joints are widespread in nature and have become a major obstacle to the safe development of underground space. Grouting reinforcement is widely adopted to reduce engineering disasters induced by joint sliding instability. In this study, direct shear tests of Polyurethane/water glass (PU/WG)-filled planar rock joints under constant normal stress conditions were carried out to investigate the effects of normal stress *σ*_n_, PU/WG layer and curing time *T* on the shear behavior, shear strength and surface damage of filled joints. The results show that PU/WG significantly enhances the peak shear strength of rock joints by 270.9% compared to the unfilled planar joints at *σ*_n_ = 2 MPa. However, the increase in *σ*_n_ reduces the improvement of shear strength for PU/WG-filled joint. With the increase of curing time, the shear strength increased rapidly within the first 10 minutes, reaching 0.2–0.31 MPa/min. After the curing time exceeds 60 min, the shear strength enhancement declines significantly. Two typical shear displacement *d*_s_ and shear stress *τ*_s_ curves are proposed, related to *σ*_n_ and filled layer. At low normal stress, such as *σ*_n_ = 2 MPa, the *τ*_s_ decreases sharply after reaching the peak shear stress for the fracture of PU/WG-rock interface. However, under high normal stress conditions, an obvious post-peak yield stage is observed, and the *τ*_s_ in the residual stage fluctuates within a certain range. High normal stress and PU/WG layer are the two necessary conditions for stick-slip in the residual stage. After shearing, the PU/WG remains largely intact on the upper surface, with few residual layers at the boundary on the lower surface. The PU/WG layer is relatively complete, with local tensile cracks, primarily V-shaped, and a few linear cracks. These findings provide valuable insights into the mechanical behavior and reinforcement effect of PU/WG filled in fractured surrounding rocks.

## 1. Introduction

Underground space creation and resources extraction are important foundations for sustainable social development. Nevertheless, in the past decades, engineering disaster such as landslide, tunnel collapse, and mine water inrush have been frequently reported worldwide [1–3]. Shear failure and slip of rock joints caused by underground excavation is considered to be the causes of disaster occurrence [4–6]. Rock joints widely distributed in the nature have become a major obstacle to the safe development of underground space. The presence of joints weakens the integrity of rock masses and leads to a significant reduction in the strength and stability [7–9]. To mitigate the instability of joints, grouting reinforcement has been widely adopted. Grouting materials are divided into two categories: organic and inorganic. Inorganic materials, such as cement, are largely applied for low cost and stable nature. However, inorganic materials containing solid particles is difficult to fill these tiny void spaces and take a long time to solidify. Organic grouting materials, represented by polyurethane, have good permeability, controllable curing time *T* and high strength, but are flammable, highly exothermic and costly [10]. Polyurethane/water glass (PU/WG) combines the advantages of organic and inorganic materials, with the superiority of flame retardant, low cost, high thermal stability, and high injectivity [11,12]. The fast solidification and high early strength meet the core requirements of fast support and immediate blocking in emergency underground engineering construction. PU/WG is gradually applied in some projects with harsh geological environment, including water-inrush sealing in deep tunnels and high-stress mine roadway stabilization. This trend has prompted researchers to pay attention to its potential to be used in complex geological conditions. Therefore, the reinforcement effect of PU/WG on rock joints is increasingly worth to be studied in depth.

Over the past decades, a series of shear tests on rock joints have been performed to investigate the influence of boundary conditions, surface topography, and fault gouge on shear behavior. Barton and Choubey (1977) proposed JRC-JCS empirical model for the shear deformation and strength characteristics of irregular rough joints, which matched well with the results of direct shear tests and has been widely used in geotechnical engineering [13,14]. Studies show that the stick-slip phenomenon is widely observed in shear tests, with joint surface roughness, normal stress, filling material properties, and shear rate being the main factors affecting it [15–17]. For the presence of soft filling materials in the joints, soft filling significantly reduces the strength of rock joints. In addition, the reduction of shear strength is closely related to the filling material properties and layer thickness [18–21]. High-strength grouting materials are filled into rock joints to increase the shear strength of rock joints. Cement slurry, the most commonly used grouting material in geotechnical engineering, is poured into rock joints to harden and form an effective cementing surface, significantly improving the shear strength and shear stiffness for rock joints. The reinforcement mechanism of cement slurry and the rock-cement interface’s mechanical behavior have been well studied [22–25]. The current research provides sufficient theoretical and empirical support for the application of cement grouting materials in rock joint reinforcement. However, in some extreme environments, traditional cement slurry is hardly to meet the grouting needs, such as rapid plugging of water inrush in a short time or advanced reinforcement of difficult-to-penetrate sandy soil strata. PU/WG slurry grouting reinforcement material can effectively solve the complex geological problems such as high permeability water flow, fractured zones and large deformation of surrounding rocks during underground space excavation due to its excellent fluidity, rapid solidification characteristics and good adhesion [26–28].

PU/WG can be effectively injected into small water channels or void spaces to seal and strengthen these channels in the event of water inrush or broken rock. As an organic-inorganic hybrid material, the basic properties, diffusion mechanism and engineering applications of PU/WG have been widely studied [29,30]. Some laboratory tests have quantitatively studied the variation of compressive strength, tensile strength and fracture toughness of PU/WG consolidation with time [29,31]. The PU/WG slurry has the characteristics of rapid solidification within 2 minutes, and the strength of the solidified body can be rapidly increased to more than 40 MPa within 2 hours [12]. Further, some improvers were added to PU/WG slurry to improve the mechanical properties of PU/WG composites to meet the construction requirements of high-performance grouting materials [32,33]. For example, Yang et al. (2017) utilized silane treatment to improve the distribution of water glass in the polyurethane matrix and increase the crosslinking density of the grouting material [27]. Current studies have shown that PU/WG slurry exhibits diffusion in rock fractures, especially in water-bearing fractures, and has superior erosion resistance in controlling water flow and preventing water inrush hazards. However, the existing research mainly focuses on the characteristics of the material itself, and the research on the mechanical behavior between PU/WG slurry and rock joints is still insufficient.

This study aims to investigate the shear behavior of planar sandstone joints filled with PU/WG slurry and to define the effect of *T* and normal stress *σ*_n_ on the reinforcement of planar joints. The direct shear tests of PU/WG- filled planar joints were conducted under conditions of constant *σ*_n_ using servo-controlled direct shear apparatus. Based on the experimental results, the evolution characteristics of the shear stress *τ*_s_ with shear displacement *d*_s_ are analyzed, and two typical shear curves are proposed for PU/WG- filled planar joints. The effects of *T* and *σ*_n_ on the improvement of shear strength were further investigated. The results may promote a deeper understanding for the grouting of fractured surrounding rock, and park our creative thinking on the prevention and control of water inrush and surrounding rock instability disasters in geotechnical engineering.

## 2. Experimental method

### 2.1. Samples preparation

**Fig 1:**
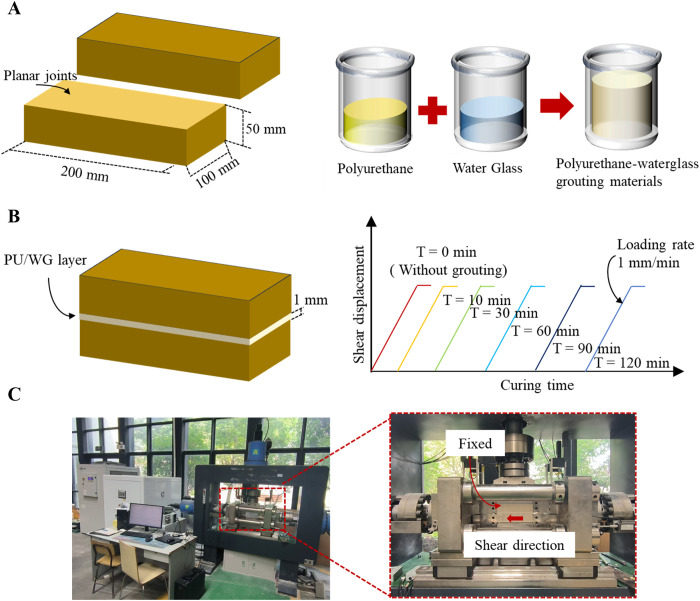
The schematic diagram for the preparation of specimen and test procedures. (A) Preparation of sandstone with planar joints. (B) Preparation of filling materials. (C) Filled sample. (D) Curing treatment. (E) Testing apparatus.

The roughness of rock joints is an important factor affecting the shear strength. However, replicating rock joints with the same roughness is challenging, and it is difficult to account for the effect of sheared debris on shear strength. Hence, in this study, planar sandstone joint samples are prepared to eliminate the interference of roughness on the evaluation of shear strength, so as to further explore the influence of PU/WG material on the joint. The sawed smooth joint samples were taken from rectangular specimens with dimensions of 200 × 100 × 100 mm. For each rectangular specimen, 200 × 100 mm surface were cut along the long side direction at the height of 50 mm of the complete sandstone, so as to obtain a couple of specimens with dimensions of 200 × 100 × 50 mm, see Fig 1(a). Subsequently, the surface was polished with fine sand-paper to form a smooth joint surface.

The PU/WG material is a non-expansive grouting material, consisting of two components: Component A mainly contains waterglass and catalyst, while Component B mainly consists of isocyanate (PAPI), polyether polyol, and plasticizer. Previous studies have shown that a volume ratio of 1:1 between components A and B is considered to be optimal [12,30]. The two halves of the specimens, measuring 200 × 100 × 50 mm, were placed in a closed mold, with 1 mm gap maintained between the two surfaces. To achieve the 1 mm filling thickness, 1 mm spacers were placed between joint surfaces before the injection of PU/WG grout, with post-curing caliper measurements confirming thickness uniformity. Then, the weighed components A and B were simultaneously poured into a beaker, stirred for 30 s at 400 rpm using a high-speed stirrer to prepare the PU/WG slurry, as shown in Fig 1(b). The blades of the agitator were close to the vessel wall to ensure uniformity of mixing. The PU/WG slurry was immediately poured into the 1 mm thick planar joints reserved in the sandstone after the stirring completed. Subsequently, the mold was vibrated to ensure that the slurry is in close contact with the joint wall. Finally, PU/WG -filled joint specimens were prepared, as depicted in Fig 1(c).

### 2.2. Test procedure

The strength of PU/WG materials increases rapidly. To study the effect of short curing time on the shear characteristics of rock joints, five curing times (e.g., 10, 30, 60, 90, and 120 min) were set (Fig 1(d)). The curing time is set at the moment when PU/WG components are mixed. Once the mixing is complete, the slurry is promptly injected into rock joint with an aperture of 1 mm. After filling, the material is allowed to stand for 2 minutes to achieve initial solidification and self-supporting capability, after which the sample is placed in the shear testing apparatus. The end of curing time is aligned with the start of shear loading. In addition, a set of planar joint samples without filling was prepared for shear testing to compare with the grouted joint specimens. The shear tests were carried out under the conditions of *σ*_n_ = 2, 4, and 6 MPa, using a servo-controlled direct shear apparatus in Fig 1(e). The apparatus is equipped with three loading units in the normal and shear directions, where the loading units in the shear direction are symmetrically arranged. The loading unit has independent control channels and can realize three types of boundary conditions: constant stress, constant displacement, and constant stiffness. All direct shear tests were performed in a controlled laboratory environment, where the temperature was precisely maintained at 25°C.

For the test, the specimen with a 1 mm thick filled layer in the middle is fixed in the shear unit. During the shearing process, the upper part of the specimen remains fixed, while the lower half is driven in the shear direction. An LVDT is installed in front of the lower part to monitor the *d*_s_. Before shearing, the *σ*_n_ is loaded to the set value at a rate of 1 MPa/min. Subsequently, the shear force is applied to the lower part of the shear unit at a constant shear rate of 1 mm/min until the *d*_s_ reaches 10 mm.

## 3. Results and discussion

### 3.1. Influence of normal stress and curing time on shear behavior

**Fig 2:**
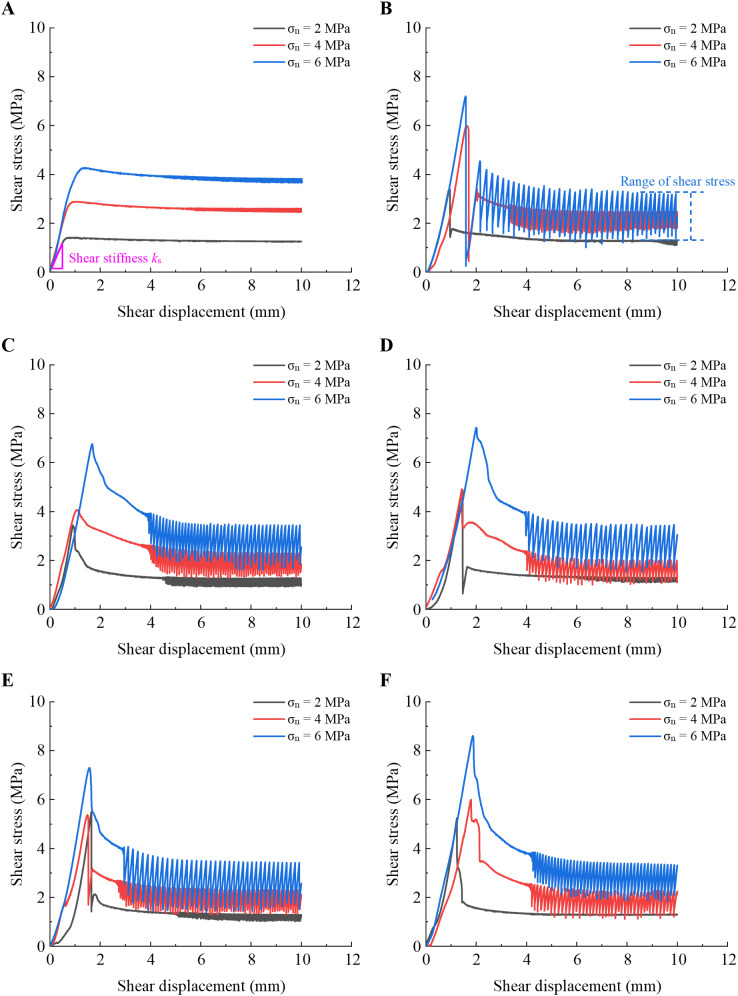
Shear stress-shear displacement curves for different curing time. (A) Unfilled planar joints. (B) *T* = 10 min. (C) *T* = 30 min. (D) *T* = 60 min. (E) *T* = 90 min. (F) *T* = 120 min.

For the convenience of analysis, the unfilled planar rock joints are set to *T* = 0 min. The mechanical results of the direct shear test at the six curing times are presented in Fig 2 (Curing time: *T* = 0, 10, 30, 60, 90, and 120 min). The test results show that the shear strength and shear behavior are heavily influenced by PU/WG filling and *σ*_n_. For the PU/WG-filled joints, a notable peak shear stress is observed due to the cementation of the joint wall by the PU/WG layer, see Fig 2(b). However, the unfilled planar rock joints exhibit a slight difference between the peak and residual shear stress (Fig 2(a)). The peak shear stress of the unfilled rock joints is significantly lower than that of the PU/WG-filled rock joints. The reinforcement effect of the PU/WG filling layer on the peak shear stress gradually stabilizes with the extension of curing time and eventually reaches a constant value. For the PU/WG-filled joints under the low normal stress (*σ*_n_ = 2MPa), a remarkable stress drop and a loud sound can be observed after the peak shear stress is reached, indicating the debonding of the PU/WG-rock interface. As the *σ*_n_ increases to 6 MPa, the high normal stress seems to inhibit the stress drop in the case of sudden fracture at *T* = 30–120 min (shown in Fig 2(c)–(f)). During the residual shear stage, the *τ*_s_ remains almost constant for the unfilled or low-stress filled rock joints. However, for the case of filled rock joints at *σ*_n_ = 6 MPa, the residual shear stress exhibits a stick-slip phenomenon, with obvious upward and downward fluctuations that increase in amplitude with *σ*_n_, as plotted in Fig 2.

**Fig 3:**
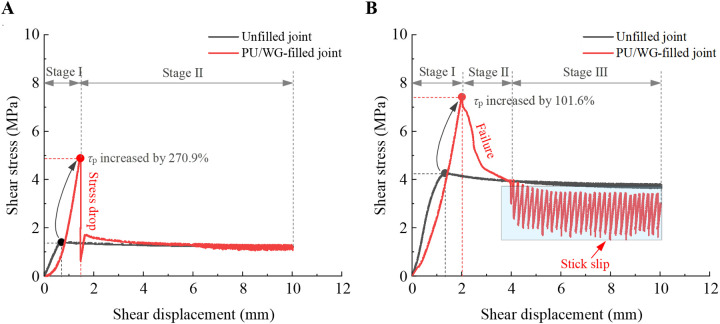
The typical shear response of low normal stress and high normal stress. (A) Example of *σ*_n_ = 2 MPa. (B) Example of *σ*_n_ = 6 MPa.

To further explore the influence of *σ*_n_ and PU/WG layer on the shear behavior of rock joints, Fig 3 shows the typical shear curves for the unfilled or filled rock joints at *σ*_n_ = 2 MPa and 6 MPa, respectively. Compared with the unfilled planar joints, the peak shear strength of PU/WG-filled joints is increased by 270.9% and 101.6% when *σ*_n_ = 2 MPa and 6 MPa, respectively. PU/WG layer significantly improves the peak shear strength for the unfilled planar joints. Two typical shear displacement-shear stress curves related to normal stress are proposed for the filled joints. At low normal stress (e.g., *T* = 60 min, *σ*_n_ = 2 MPa), the shear displacement-shear stress curve can be divided into two stages, i.e., stage Ⅰ and stage Ⅱ. In stage Ⅰ, the *τ*_s_ increases linearly with the *d*_s_ and eventually reaches the peak of the shear strength (Fig 3(a)). In stage Ⅱ, the *τ*_s_ drops sharply due to the fracture of the PU/WG-rock interface. As the influence of the stress drop caused by the fracture disappears, the joint enters a stable shear sliding, during which the residual shear stress remains constant and is essentially the same as that of the unfilled joint. Fig 3(b) shows the evolution of *τ*_s_ with *d*_s_ for the filled and unfilled rock joints under the high normal stress condition. At high normal stress (e.g., *T* = 60 min, *σ*_n_ = 6 MPa), the shear displacement-shear stress curve evolves through three stages. The change in *τ*_s_ is similar to that at low normal stress in stage Ⅰ, showing a rapid increase in a linear manner. In stage Ⅱ, the high normal stress restrains the drastic reduction in *τ*_s_ caused by the fracture of the bonding surface and shows obvious shear yield. During stage III, a stick-slip phenomenon occurs in the residual stage with the shearing. In the stick-slip state, the residual shear stress varies in a certain stress range, and its peak value is close to the residual shear stress of the unfilled joint.

The *σ*_n_ and filling layer have a significant impact on the interface contact area and contact pressure, which further affect the shear behavior of joints. At low normal stress, the filling layer significantly enhances the shear strength of joints, casing the *τ*_s_ at failure to exceed the set *σ*_n_ greatly and triggering severe localized stress concentrations. As brittle fracture occurs at the PU/WG-rock interface, the accumulated energy is rapidly released, resulting in a sharp decrease in *τ*_s_ occurs. However, under high normal stress, the increased interface contact area and pressure enhance the adhesion strength of the PU/WG-rock interface. This promotes the plastic deformation of the PU/WG layer, allowing the joint to gradually yield rather than fracture abruptly after reaching the peak *τ*_s_. Sandstone is a brittle material, and in contrast, PU/WG consolidation exhibits better toughness and viscoelasticity. The difference in the properties of the two materials leads to inconsistent interactions for the interface, causing fluctuations in *τ*_s_ and stick-slip phenomena. When *σ*_n_ = 2 MPa, the PU/WG filling layer lacks sufficient deformation to support the energy accumulation and release required for stick-slip, so the stick-slip phenomenon is hardly observed in the residual stage. However, under high normal stress, e.g., *σ*_n_ = 6 MPa, the PU/WG filling layer can realize large deformation, resulting in the more obvious stick-slip phenomenon. In addition, the surface of sandstone is rough, while the PU/WG grouting layer presents a relatively smooth surface. This difference in surface roughness may lead to the inhomogeneity of the interface contact, inducing local adhesion and slip during the shearing process.

### 3.2. Shear strength enhancement characteristics with curing time

**Fig 4:**
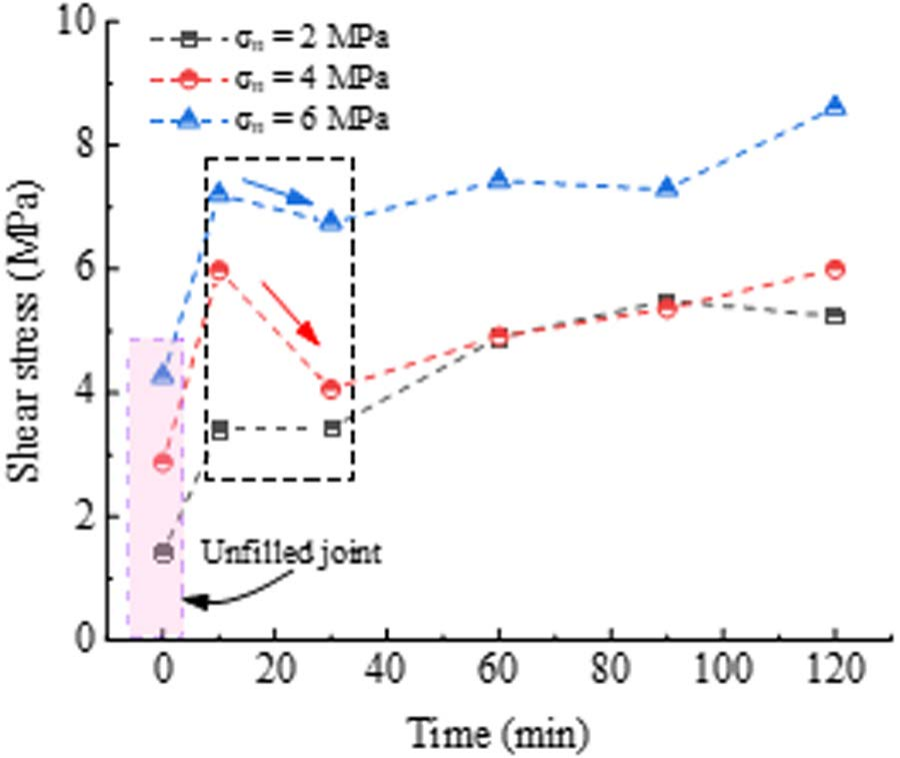
Variations of shear strength with curing time and normal stress.

Fig 4 demonstrates the peak shear strength evolution with increasing curing time under different normal stress conditions. For the PU/WG-filled planar joint, the shear strength is significantly improved owing to the reinforcement effect of the filling material. Compared to the unfilled joint, the shear strength increased by 140.6%, 107.8%, and 68.6% at *σ*_n_ = 2, 4, and 6 MPa, respectively, when *T* = 10 min. It is worth noting that the shear strength is slightly lower at *T* = 30 min compared to *T* = 10 min. For the case of *σ*_n_ = 6 MPa and *T* = 10 min, the shear strength was 7.2 MPa, decreasing to 6.8 MPa at *T* = 30 min. At short curing time (e.g., *T* = 10 min), the PU/WG layer has not sufficiently solidified and hardened. Hence, under the action of *σ*_n_, the insufficiently solidified slurry in the PU/WG-rock interface enters the void space of the sandstone, increasing the contact area and bond between the PU/WG layer and the sandstone surface. As the curing time exceeds 60 min, the effect of curing time on the shear strength becomes relatively limited, with only a slight increase. For instance, when *σ*_n_ = 2 MPa, the shear strength increases by 0.6 MPa and 0.3MPa as the curing time is extended from 60 min to 90 min and 120 min, respectively. This is because the consolidation of the PU/WG layer is essentially completed at *T* = 30–120 min, resulting in a gradual strength increase with longer curing time.

**Fig 5:**
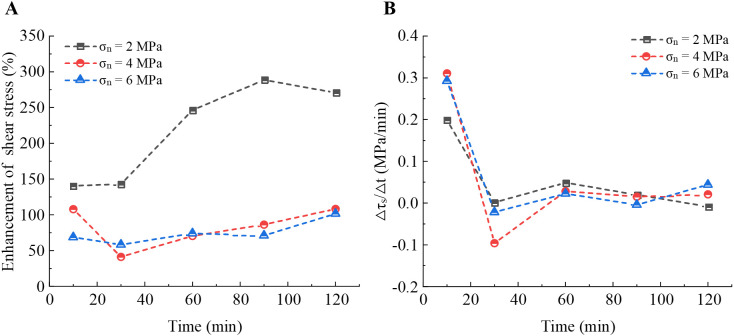
Effect of PU/WG on shear strength of planar joints. (A) Enhancing effect of PU/WG on shear strength. (B) Shear stress increment with unit time.

The influence of *σ*_n_ on peak shear strength is significant. The results show that the enhancement effect of the PU/WG layer on shear strength is better under the lower normal stress (*σ*_n_ = 2 MPa) compared to 4 MPa and 6 MPa. As shown in Fig 5(a), the enhancement of shear strength reaches 140.6% to 270.9% for *T* = 10–120 min under *σ*_n_ = 2 MPa, while it is 70.6% to 108.3% and 58.3% to 101.6% under *σ*_n_ = 4 MPa and 6 MPa, respectively. The peak shear strength of low normal stress primarily depends on the cementation of the PU/WG layer and the joint wall. As the *σ*_n_ increases, the required shear strength for unfilled planar joints increases. Although, the increased contact area is helpful to improve the shear strength, the PU/WG layer may experience localized loosening with the PU/WG-rock interface under high normal stress, resulting in a decrease in shear strength [20,34].

To quantitatively evaluate the enhancement of filling layer on shear strength with time, the shear strength growth in unit time is shown in Fig 5(b). The results indicate a rapid increase in the early stage, reaching 0.2–0.31 MPa/min within the first 10 minutes for the *σ*_n_ = 2, 4, and 6 MPa. This is attributed to the rapid hardening reaction of the PU/WG material in the initial stage, where the chemical reaction between polyurethane and water glass rapidly forms a high-strength cementation layer. Additionally, there is a notable exothermic reaction occurring during the initial phase of the PU/WG interaction. This exothermic process raises the local temperature of the reaction system, thereby accelerating the solidification of PU/WG and leading to a rapid increase in its strength within the first 10 minutes. As the exothermic reaction weakens, the system temperature gradually stabilizes, resulting in a slower rate of strength development. However, during 10−30 minutes, the shear strength increases slowed significantly to 0.0016, −0.096, and −0.022 MPa/min for the *σ*_n_ = 2, 4, and 6 MPa, respectively, due to the adsorption and local adjustment in the filling layer. As the curing time reached 60−120 minutes, the shear strength shows a slight growth ranging from −0.0084 to 0.049 MPa/min, despite ongoing hardening within the PU/WG layer. The strengthening effect on shear strength had slowed down substantially. As the hardening degree improved, the permeability and cementation between the slurry and the rock surface gradually weakened, which directly impacted further shear strength growth.

### 3.3. Evolution of residual shear strength and shear stiffness

**Fig 6:**
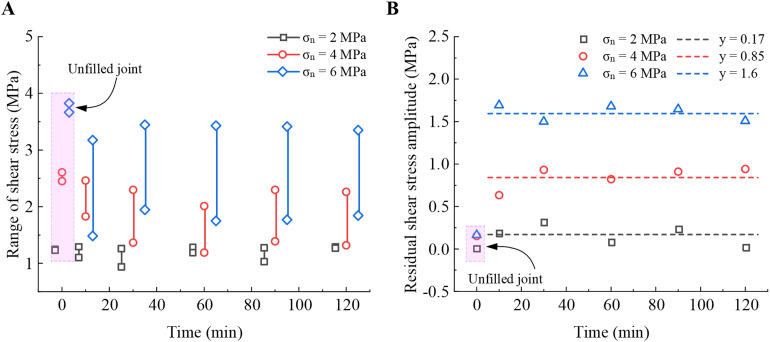
Effect of PU/WG on shear stress of planar joints at residual stage. (A) Range of shear stress under different curing time. (B) Residual shear stress amplitude.

The possible stick-slip phenomenon of filled joints during the residual stage of shear is observed in Fig 2, shown by the regular fluctuation of the residual shear stress within a certain range. Fig 6 demonstrates the peak and valley values of the *τ*_s_ variations in the residual stage under different curing times to analyze the effect of the filling layer on the shear behavior of planar sandstone joints. For unfilled joints or filled joints at lower normal stress (2 MPa), stick-slip hardly occurs, and the *τ*_s_ remains almost constant during the residual stage. As shown in Fig 6(b), the average amplitude is 0.17 MPa at *σ*_n_ = 2 MPa for filled joints, while it is 0.1 MPa for unfilled joints. However, the residual shear stress amplitude is 0.85 MPa and 1.6 MPa at *σ*_n_ = 4 MPa and 6 MPa, respectively, 5 and 9.4 times higher than at *σ*_n_ = 2MPa.The amplitude of the residual shear stress increases with the increase of *σ*_n_. This indicates that high normal stress provides conditions for stick-slip occurrence by increasing contact pressure and adhesion. In this study, high normal stress and PU/WG layer are essential for the occurrence of the stick-slip phenomenon. Additionally, curing time has a negligible impact on the amplitude of *τ*_s_ during the residual stage.

**Fig 7:**
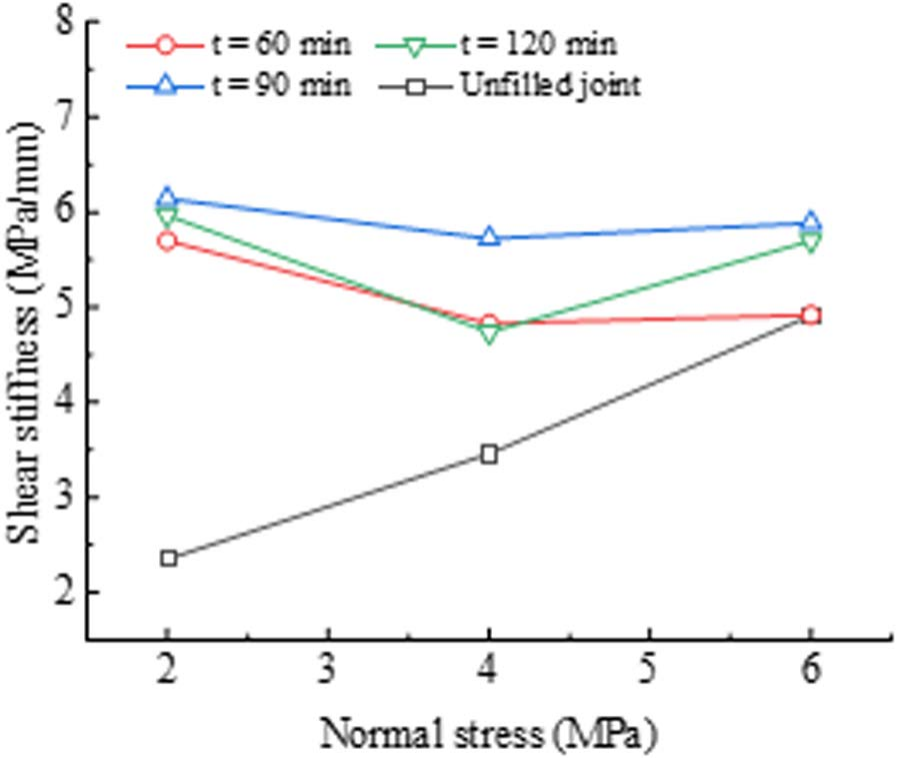
Effect of PU/WG on shear stiffness for different normal stress.

As shown in Fig 7, the shear stiffness is influenced by *σ*_n_ and PU/WG layer. The shear stiffness is defined as the slope of *τ*_s_/*d*_s_ in the linear elastic stage (see Fig 2(a)). For unfilled joints, the shear stiffness increases linearly from 2.3 to 4.9 MPa/mm as *σ*_n_ rises from 2 to 6 MPa. This indicates that the shear stiffness is significantly dependent on the applied *σ*_n_. Higher normal stress inhibits the improvement of the PU/WG layer on the shear stiffness for joints. At *σ*_n_ = 2 MPa, the shear stiffness of filled joints reaches 5.7–6.1 MPa/mm at *T* = 60–120 min, about three times that of unfilled joints (2.3 MPa/mm). When the *σ*_n_ is 6 MPa, the shear stiffness ranges from 4.9 to 5.9MPa/mm for filled joints, while the unfilled joint is 4.9 MPa/mm. The PU/WG layer only slightly increases the shear stiffness when *σ*_n_ = 6 MPa, which may explain the difference in post-peak yielding behavior due to different normal stresses in Fig 3. With increasing *σ*_n_, the shear stiffness decreases for filled joints. For example, at *T* = 60 min, the shear stiffness decreased from 5.7 to 4.9 MPa/mm as *σ*_n_ rose from 2 to 6 MPa. High normal stress leads to local deformation and loosening. This occurs because the shear stiffness during the initial elastic stage is primarily influenced by the deformation of the PU/WG layer and the PU/WG-rock interface. Under high normal stress conditions, the PU/WG layer is triggered to strain localization, forming micro-crack network. Additionally, due to the inconsistent deformation of the PU/WG layer and the rock matrix, the high normal stress promotes the development of loosening zones at the PU/WG-rock interface. These factors further contribute to the observed reduction in shear stiffness under a higher normal stress.

### 3.4. Damage characteristics of PU/WG-filled planar rock joint

**Fig 8:**
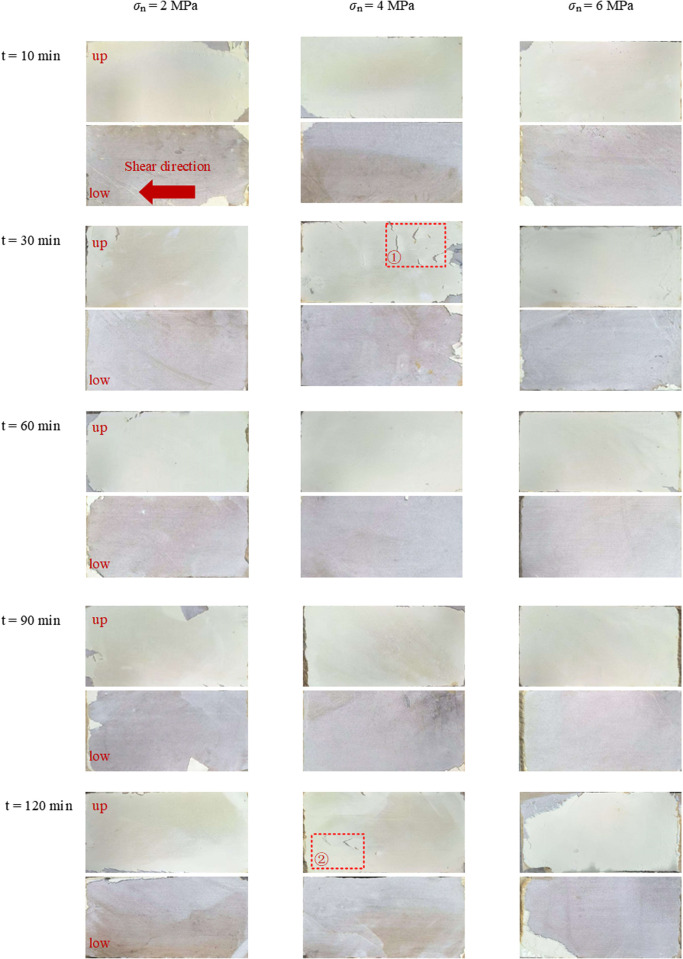
Surface morphology of PU/WG filled joint samples after shear tests.

After the shear test, the surface morphology of the PU/WG-filled joint specimen is shown in Fig 8. The PU/WG forms a smooth and dense filling layer after solidification, while the polished sandstone still maintains significant roughness and porosity. Furthermore, the difference in material properties between the filling layer and sandstone matrix leads to incompatible deformation during shear, causing the PU/WG-rock interface bond to be unstable and prone to fracturing. During shearing, the upper part of the specimen is fixed, while the lower part is sheared at a constant rate of 1 mm/min. After shear damage, the PU/WG layer remains strongly bonded to the upper sandstone surface, but only a few of the residual PU/WG layer exists on the lower sandstone surface at the boundary. The phenomenon of non-uniform failure primarily arises from the asymmetric distribution of interface stress, which is caused by the directional movement of the upper and lower specimens during the shear process. When the lower sandstone slides horizontally, it experiences a higher shear strain rate and stress concentration, leading to preferential bonding failure at the PU/WG-rock interface.

**Fig 9:**
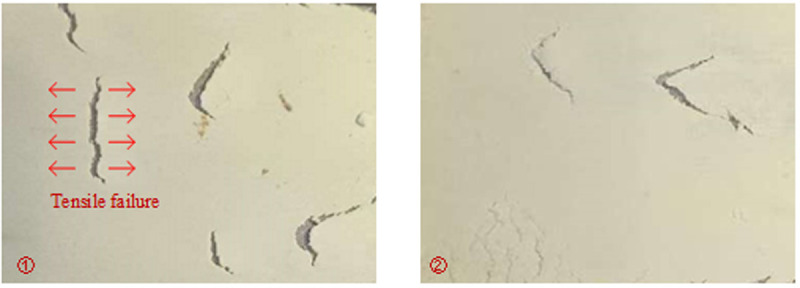
Damage characteristics of PU/WG layer.

Fig 9 shows the partial damage characteristics of the filling layer. Despite the overall good integrity of the consolidated layer on the sandstone surface, localized tension cracks are observed. The morphology of these cracks exhibits mainly in a V-shaped pattern, accompanied by a few linear patterns. Notably, the tip direction of the V-shaped crack is aligned with the shear direction of the sample. This crack may develop as a result of stress concentration during the shear process, beginning at a weak point on the surface. When the local tensile stress exceeds the tensile strength of the material, a microcrack initiates at this location and propagates in the direction of the maximum principal tensile stress, at an angle of approximately 45° to the shear direction [35–37].

## 4. Conclusions

In this study, direct shear tests on polyurethane/water glass (PU/WG)-filled planar rock joints were conducted under constant normal stress. The effects of normal stress *σ*_n_, PU/WG layer, and curing time *T* on shear behavior, shear strength, and surface damage were analyzed, with unfilled planar sandstone joints as controls. The main conclusions are as follows.

The PU/WG layer and *σ*_n_ significantly impact joint shear behavior. The PU/WG-rock interface increased peak shear strength by up to 270.9%. High normal stress inhibits the sharp reduction of shear stress caused by joint fracture, and promotes stick-slip in the residual phase. As the curing time of the filled layer increased, peak shear strength increased rapidly, reaching 0.2–0.31 MPa/min within the first 10 minutes. However, the strengthening effect diminished with the further increase of curing time. The enhancement of shear strength was significantly better in the low normal stress, about 2.7 times that of high normal stress. High normal stress and PU/WG layer are required for stick-slip to occur during the residual phase, and the range of residual shear stress increased with *σ*_n_. High stress diminished the enhancement of shear stiffness. The improvement of shear stiffness reaches 3 times at *σ*_n_ = 2 MPa but is only slightly enhanced at 6 MPa. After shearing, the PU/WG-filled layer is nearly fully bonded to the upper surface of the sandstone, with only a slight residual filling layer remaining on the lower surface at the boundary. The PU/WG-filled layer on the upper surface of the sandstone remains relatively intact, accompanied by some localized tension cracks, dominated by V-shaped cracks, and a few linear cracks. This study enhances our understanding of the mechanical behavior of PU/WG-filled plane rock joints and provides essential theoretical support for the rapid reinforcement of jointed rock masses in complex geological environments, such as deep tunnels and mining roadways.

Although PU/WG slurry is effective for reinforcing complex geological conditions like fractures and large deformations, this study does not address the influence of rock joint roughness on its mechanical behavior. Additionally, the long-term durability of the slurry in extreme environments, such as freeze-thaw cycles or high temperatures, remains uncertain. Future research will aim to investigate the material response to joint roughness, as well as its durability and temperature sensitivity under multi-field coupling conditions.
